# Obesity May Not Be Associated with 28-Day Mortality, Duration of Invasive Mechanical Ventilation and Length of Intensive Care Unit and Hospital Stay in Critically Ill Patients with Severe Acute Respiratory Syndrome Coronavirus-2: A Retrospective Cohort Study

**DOI:** 10.3390/medicina57070674

**Published:** 2021-06-29

**Authors:** Sjaak Pouwels, Dharmanand Ramnarain, Emily Aupers, Laura Rutjes-Weurding, Jos van Oers

**Affiliations:** Department of Intensive Care Medicine, Elisabeth-Tweesteden Hospital, Hilvarenbeekseweg 60, P.O. Box 90151, 5000 LC Tilburg, The Netherlands; d.ramnarain@etz.nl (D.R.); e.aupers@etz.nl (E.A.); l.weurding@gmail.com (L.R.-W.); jah.vanoers@etz.nl (J.v.O.)

**Keywords:** severe acute respiratory syndrome coronavirus-2, SARS-CoV-2, obesity, length of stay, intensive care unit, invasive mechanical ventilation

## Abstract

*Background and Objectives:* The aim of this study was to investigate the association between obesity and 28-day mortality, duration of invasive mechanical ventilation and length of stay at the Intensive Care Unit (ICU) and hospital in patients admitted to the ICU for SARS-CoV-2 pneumonia. *Materials and Methods:* This was a retrospective observational cohort study in patients admitted to the ICU for SARS-CoV-2 pneumonia, in a single Dutch center. The association between obesity (body mass index > 30 kg/m^2^) and 28-day mortality, duration of invasive mechanical ventilation and length of ICU and hospital stay was investigated. *Results:* In 121 critically ill patients, pneumonia due to SARS-CoV-2 was confirmed by RT-PCR. Forty-eight patients had obesity (33.5%). The 28-day all-cause mortality was 28.1%. Patients with obesity had no significant difference in 28-day survival in Kaplan–Meier curves (log rank *p* 0.545) compared with patients without obesity. Obesity made no significant contribution in a multivariate Cox regression model for prediction of 28-day mortality (*p* = 0.124), but age and the Sequential Organ Failure Assessment (SOFA) score were significant independent factors (*p* < 0.001 and 0.002, respectively). No statistically significant correlation was observed between obesity and duration of invasive mechanical ventilation and length of ICU and hospital stay. *Conclusion:* One-third of the patients admitted to the ICU for SARS-CoV-2 pneumonia had obesity. The present study showed no relationship between obesity and 28-day mortality, duration of invasive mechanical ventilation, ICU and hospital length of stay. Further studies are needed to substantiate these findings.

## 1. Introduction

In December 2019, the first patients suffered from an unknown disease-entity causing pneumonia-like symptoms. Later on, weeks after the first deaths, the novel strain of a virus, severe acute respiratory syndrome coronavirus-2 (SARS-CoV-2), was identified. SARS-CoV-2 spread rapidly across the globe causing major health catastrophes and widespread panic, social unrest and economic instability of which the long-term consequences are yet to be determined [1,2,3]. Unfortunately, to date, the rate of newly diagnosed patients is still rising, [1] In several papers, there seem to be different factors that might be associated with an increased risk of hospitalization and mortality in patients with SARS-CoV-2. Among them are advanced age (>60 years), obesity (body mass index (BMI) > 30 kg/m^2^), diabetes, hypertension, cardiovascular disease, as well as a history of smoking and chronic obstructive pulmonary disease (COPD) [1,2,3,4,5,6]. In several clinical reports, obesity was determined as an independent risk factor for SARS-CoV-2 increased severity of the disease and death [7,8,9,10]. In a recent study conducted by Simonnet et al. [10], there was a high prevalence of obesity among patients with SARS-CoV-2 requiring invasive mechanical ventilation. Moreover, severe obesity (defined as (BMI)> 35 kg/m^2^) was significantly associated with the need for invasive mechanical ventilation [10]. Similar results were found in the clinical report of Caussy et al. [11]. However, there is currently no evidence of the effects of overweight and obesity on the duration of invasive mechanical ventilation, hospital and/or intensive care unit (ICU) stay and 28-day mortality.

The primary aim of the present study was to investigate the association between obesity and 28-day mortality. Secondly, the association between obesity and duration of invasive mechanical ventilation and length of stay (LOS) at the ICU and hospital in patients admitted to the ICU for SARS-CoV-2 was investigated.

## 2. Materials and Methods

### 2.1. Study Design and Patient Enrolment

In this single-center retrospective observational cohort study, we enrolled all patients that were admitted to the ICU for SARS-CoV-2 pneumonia in the Elisabeth-Tweesteden Hospital in Tilburg in the Netherlands between 2 March and 5 June 2020. Patients were diagnosed according to the criteria that were set up by the World Health Organization interim guidance [1,2]. Patients were suspected of a SARS-CoV-2 pneumonia if one or more of the following symptoms were present (on either a conventional chest X-ray or a computed tomography (CT) scan) and one or more of the following symptoms were present: dyspnea, increased respiratory insufficiency, decreased blood oxygen saturation and the need for supportive oxygen therapy [1,2]. Throat swab samples were obtained from all patients at admission and were tested using a real-time reverse transcriptase-polymerase chain reaction assay (RT-PCR) to identify a SARS-CoV-2 infection [1,2]. In case of a high suspicion based on clinical judgement or computed tomography (CT) scan and multiple negative tests, deep bronchial aspirates and feces tests were performed to determine the diagnosis [1,2]. Patients with a pneumonia were excluded if no SARS-CoV-2 could be detected by PCR or if SARS-CoV-2 pneumonia was present.

This study was performed in accordance with the ethical standards as laid down in the 1964 Declaration of Helsinki and was conducted according to the Strengthening the Reporting of Observational studies in Epidemiology (STROBE) statement [3]. The Medical Ethical Committee of Elisabeth-Tweesteden hospital in Tilburg, the Netherlands, approved this study and waived the need for informed consent (protocol number: L0977.2020).

### 2.2. Outcomes

The primary outcome of this study was 28-day mortality in critically ill patients with SARS-CoV-2. Secondary outcomes were the length of hospital stay, length of ICU stay and the duration of invasive mechanical ventilation. Length of hospital stay and length of ICU stay were defined as the total duration of hospital or ICU admittance for a SARS-CoV-2 infection, counting the ventilator days, which are the total number of days that patients were on a mechanical ventilator, assessed the duration of invasive mechanical ventilation. The following patient characteristics were analyzed: gender, age, height, length, BMI, and the presence of comorbidities.

### 2.3. Data Collection and Statistical Analysis

Clinical data and microbiological and laboratory results were prospectively collected on a daily basis in patients enrolled in the study. Three trained physicians (S.P., D.R., J.v.O.) collected and reviewed the epidemiological data, past medical history, treatments, clinical data and outcomes for all consecutive patients from their admission to 5 June 2020.

Because of the retrospective and exploratory nature of this study, no formal sample size and power calculation was done. All non-normally distributed data (Kolmogorov–Smirnov test *p* < 0.05) are expressed as median (with interquartile range, IQR) or as number of patients (percentage) where appropriate. Patient characteristics and outcomes were compared using a Mann–Whitney U test for continuous variables and chi-square test for categorical variables. The cumulative survival was analyzed by applying the Kaplan–Meier curves, and differences in mortality were compared with the log rank test. Univariate and multivariate Cox regression proportional hazards regression analyses were conducted to study the effects on 28-day mortality. The relation between obesity and duration of invasive mechanical ventilation, LOS ICU and hospital was analyzed by correlation analysis. All tests were two-sided and a *p*-value < 0.05 was considered statistically significant. All data were analyzed using a statistical software package (SPSS Inc., version 24, Chicago, IL, USA)

## 3. Results

### 3.1. Descriptive Characteristics of the Patients

**Figure 1:**
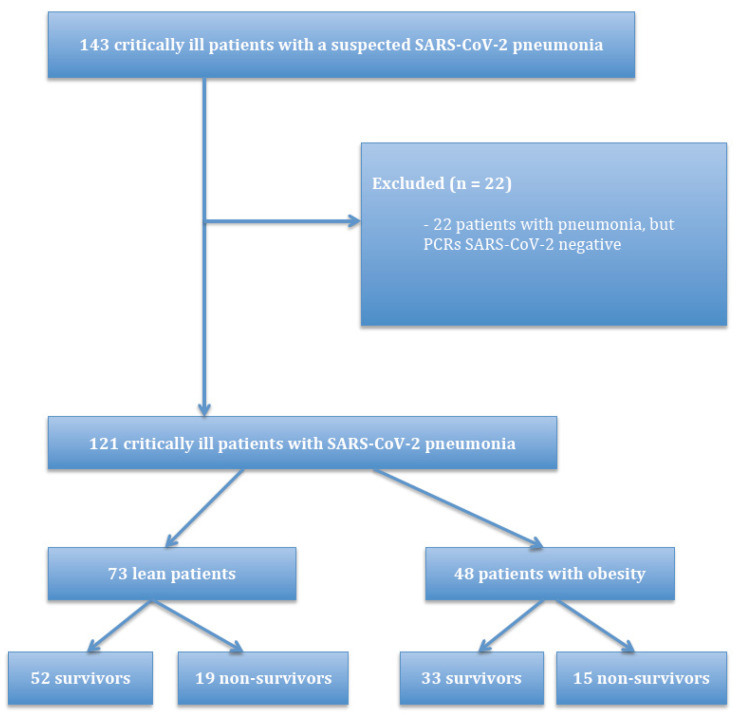
Patient inclusion flow diagram.

A total of 143 patients were admitted to the ICU with a suspected SARS-CoV-2 pneumonia during the study period. In 121 patients with pneumonia, SARS-CoV-2 was confirmed by RT-PCR. The patient flow diagram shows the flow of patients along with the primary endpoint of 28-day survival (Figure 1).

Demographics and clinical characteristics of the 121 selected patients are shown in Table 1. A total of 116 patients were septic, and 12 patients were in septic shock, according to the Sepsis-3 definitions. The 28-day all-cause mortality was 28.1%. Median duration of invasive mechanical ventilation was 14 days, and ICU and hospital LOS were 16 and 20 days, respectively. Patients were divided in two groups: patients with and without obesity. Forty-eight patients had obesity (33.5%). Both groups were comparable except for older age in the group of patients without obesity. There were also no significant differences in inflammatory biomarkers, white blood count (WBC) and C-reactive protein (CRP) between patients with and without obesity.

### 3.2. Association between Obesity and 28-Day Mortality

**Figure 2:**
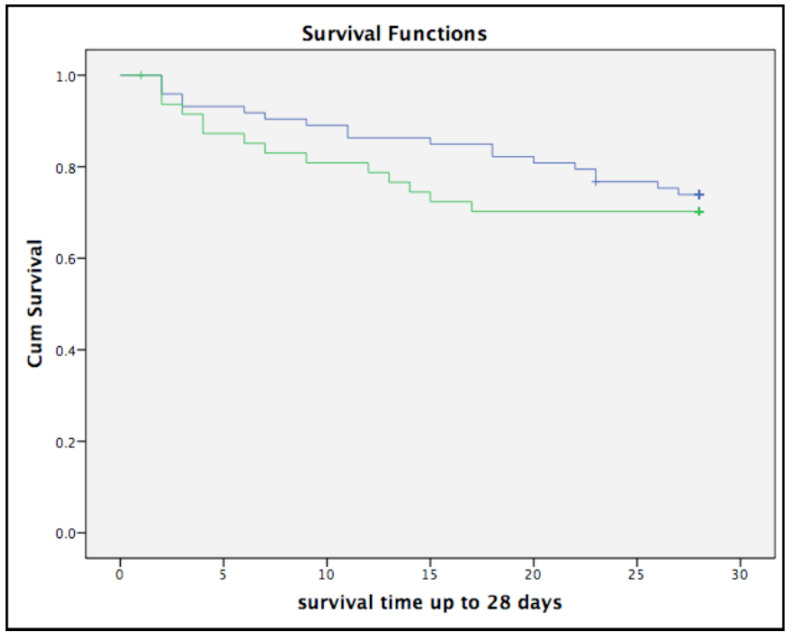
Kaplan–Meier curve 28-day survival. Legend: Blue line: BMI ≤ 30 kg/m^2^, green line: BMI > 30 kg/m^2^. Log rank *p* = 0.545.

All-cause 28-day mortality in all patients was 28.1%. There were no significant differences in 28-day mortality between patients with and without obesity (*p* = 0.545) (Figure 2). Obesity made no significant contribution in univariate and multivariate Cox regression analysis for prediction of 28-day mortality (Table 2). Age (HR 1.11 (1.05–1.17), *p* < 0.001) and SOFA (Sequential Organ Failure Assessment) score (HR 1.37 (1.12–1.67), *p* = 0.002) were significant independent factors in a multivariate model for prediction of 28-day mortality. Table 3 and Table 4 show univariate and multivariate analyses for males and females separately. In males (Table 3), both SOFA score (HR 1.36 (1.08–1.73), *p* = 0.01) and age (HR 1.12 (1.05–1.20), *p* = 0.001) remain significant independent factors for prediction of 28-day mortality, whereas in females both are not significant. 

### 3.3. Association between Obesity and Duration Mechanical Ventilation, LOS ICU and Hospital

No statistically significant correlation was observed between BMI and number of days on the ventilator (Spearman’s rho 0.009, *p* = 0.925). No significant correlation between BMI and number of ICU days (Spearman’s rho −0.053, *p* = 0.563) and no significant correlation between BMI and number of days in hospital (Spearman’s rho −0.093, *p* = 0.328) were found/observed.

## 4. Discussion

The primary aim of our study was to investigate the association between obesity and 28-day mortality. Secondary outcomes were the association between obesity and duration of invasive mechanical ventilation, length of stay (LOS) at the ICU and hospital in patients admitted to the ICU for SARS-CoV-2. In our study we found no relationship between obesity and 28-day mortality in a cohort of patients admitted at the ICU department for SARS-CoV-2. Additionally, in our study there was no relationship between obesity and ICU length of stay, hospital length of stay and ventilator days. Only age and SOFA were significant independent factors associated with 28-day mortality.

Worldwide changes in lifestyle, consumer markets and urbanization are important causes of the obesity pandemic. Since 1975, obesity incidence and prevalence numbers have been rising significantly, according to the World Health Organization (WHO) [4,5,6].

To date it has been estimated that approximately 1.9 billion adults are overweight, and secondly, there are approximately 650 million people with obesity [5,6]. Comparing these numbers with the 1970s, it indicates a rise of 300% [5,6]. Of course we also have to take into account that the world population also grows, but still there is a global prevalence of obesity of 37% compared with 27% back in the 1970s [5,6]. It is no surprise that the obesity pandemic has a high impact on health care providers globally, and with the SARS-CoV-2 pandemic, it has been put into a “new” perspective [5,6]. In general, patients with obesity have an increased risk of developing type 2 diabetes mellitus (T2DM), obstructive sleep apnea syndrome (OSAS) and numerous of cardiovascular related diseases [4,7,8,9].

Recently both Simonnet et al. [10] and Caussy et al. [11] showed that there is a high prevalence of patients with obesity and severe obesity in patients with SARS-CoV-2 who are in need of invasive mechanical ventilation. Both studies were performed in France and showed a different distribution in SARS-CoV-2 patients among different BMI categories. Caussy et al. [11] pointed out that in their study there was a lower prevalence of patients with a BMI ≥ 35 kg/m^2^ than in the study done by Simonnet et al. [10] (respectively, 11.3% and 28.2%). Same goes for the need for invasive mechanical ventilation (Caussy et al. [11] 58.4% compared with 68.6% in the study of Simonnet et al. [10]). This might be due to a difference in the prevalence of obesity in several regions and also due to the kind of strategy that is used in the treatment of these SARS-CoV-2 patients, e.g., very fast intubation or a more conservative treatment schedule [11]. It also needs to be mentioned that both studies were done in patients in the general ward. In our study, we only included ICU patients.

In the study by Caussy et al. [11], there was a higher use of high-flow oxygen therapy through a nasal cannula such as OptiflowTM (56%). In the study by Simonnet et al. [10], this was not reported. Our own study consisted of 10 patients (8.3%) that needed high nasal flow oxygen therapy. Recent studies done by Gupta et al. [12] and Ebinger et al. [13] indicated that obesity is associated with SARS-CoV-2 and even with increased mortality according to Gupta et al. The study by Czernichow et al. [14] showed that mortality doubles among different classes of obesity. In this study, almost 6000 patients were included, and mortality was significantly increased in people with obesity with the following OR in BMI 30–35, 35–40 and >40 kg/m^2^: 1.89 (95%CI 1.45–2.47), 2.79 (1.95–3.97) and 2.55 (1.62–3.95). Similar results were found in the study by AbdelMassih. [15] There is also a higher non-respiratory complication rate in patients with obesity, in particular, renal failure and shock [16]. Here it has to be taken into account that the studies by Czernichow et al. [14] and AbdelMassih [15] were done in a population on the general ward, and both studies were not specific for the ICU population. 

With the SARS-CoV-2 pandemic and the identification of obesity as potential risk factor according to several reports [10,11,12,13], we are basically handling a “double edged sword”. In the study of Caussy et al. [11], the authors state that patients with obesity might have an increasing disease severity; however, no severity score was used to assess the matter. Regarding the pathophysiology, there might be an association between SARS-CoV-2 and obesity; however, the potential mechanisms are not yet understood. In the past, obesity has been described as an independent predisposition factor for H1N1 pulmonary infection [17]. Abdominal obesity is known to possibly significantly impair ventilation in the lower parts of the lungs, resulting in reduced oxygen saturation [18]. Furthermore, it needs to be stated that inflammation is closely associated with obesity and might impair the immune response [19]. There is an inflammatory reaction initiated when adipose tissue is expanded, which causes infiltration of immune cells [20]. Studies have shown increased levels of tumor necrosis factor-alpha (TNF-α), expression of ET-1, IL-1, IL-6 and reactive oxygen species (ROS) that can be produced by adipose tissue [21]. This state of chronic low-grade inflammation can lead to a dysregulated immune response in patients with obesity and SARS-CoV-2 and can have effects on the lung parenchyma and bronchi [19,22]. However, in our study, inflammatory biomarkers were not significantly different between patients with and without obesity. It has to be taken into account that the pathophysiological influences of obesity and COVID-19 comprise multifactorial mechanisms, which are still not fully understood. In a comprehensive review done by Sanchis-Gomar et al. [23], it has been stated that SARS-CoV-2 binds with angiotensin-converting enzyme 2 receptors on the cell surface. These receptors are more prevalent in adipose tissue, which might indicate that patients with obesity may be more susceptible to a SARS-CoV-2 infection with a possibly more severe clinical course. In a lot of epidemiological studies, obesity is a significant risk factor for mortality and morbidity of a SARS-CoV-2 infection [24,25]. However it has been shown by Kooistra et al. that patients with a BMI > 30 kg/m^2^ do not have a more severe clinical course in the ICU than patients with a BMI lower that 30. The same is the case for their immune response. Also similar to our study results, there was no significant difference between ICU-related outcome measurements (ICU length of stay, days of mechanical ventilation and 40-day mortality) between the two BMI groups. Thus, this is a part of the “obesity paradox” that clearly needs more research to elucidate possible mechanisms [26].

### Limitations

There are some limitations that need to be addressed. Firstly, this was a small retrospective single-center study in which the results are hypothesis generating but cannot be used to determine a cause-and-effect relationship between obesity and SARS-CoV-2. Secondly, selection bias needs to be taken into account since our study only investigated ICU patients with SARS-CoV-2 pneumonia. Thirdly when comparing our study with other studies in the literature (Simonnet et al. [10] and Caussy et al. [11]), the median BMI in our study was lower, which might have impacted our results. Fourthly, since this study was a small retrospective study, the cross effects between different predictors could not be determined because of our sample size. Additionally, it needs to be mentioned that this retrospective study was purely focused on the patients with COVID-19 admitted to the ICU. We investigated the effects on clinical parameters in patients with obesity and COVID-19 admitted to the ICU. This is a very small subset of the COVID-19 population. Since the small number of patients might indicate that our study is underpowered, we do have to take into account that de study by Kooistra et al. [26] substantiated our findings. They showed that only that there is no clinical difference between BMI groups in ICU admitted COVID-19 patients, but there is also no difference in cytokine and immune reaction in these patients. This is probably due to the “obesity paradox”, and to further elucidate this in ICU patients with COVID-19 and obesity, larger studies are indeed needed. 

## 5. Conclusions

No relation was found between obesity and 28-day mortality, ICU length of stay, hospital length of stay and ventilator days in a cohort of 121 patients admitted at the ICU department for SARS-CoV-2. Only age and the SOFA were significant independent factors associated with 28-day mortality.

## Figures and Tables

**Table 1:** Clinical characteristics of patients with COVID-19 pneumonia at baseline with regard to body mass index.

	Total	Non-Obese (BMI ≤ 30 kg/m^2^)	Obese (BMI > 30 kg/m^2^)	*p* Value
	(n = 121)	(n = 73)	(n = 48)	
Age (years) (median, IQR)	68 (60–74)	71 (63–75)	65 (56–71)	0.003
Male gender (N, %)	91 (75.2%)	58 (79.5%)	33 (68.8%)	0.182
**Pre-existing comorbidities (N, %)**				
Hypertension	37 (30.6%)	21 (28%)	16 (33.3%)	0.594
Congestive heart failure	23 (19%)	11 (15.1%)	12 (25%)	0.173
COPD	20 (16.5%)	10 (13.7%)	10 (20.8%)	0.301
Diabetes mellitus	31 (25.6%)	16 (21.9%)	15 (31.3 %)	0.250
Cerebrovascular disease	6 (5%)	4 (5.5%)	2 (4.2%)	0.745
Malignancy	18 (14.9%)	11 (15.1%)	7 (14.6%)	0.942
Chronic renal disease	6 (5%)	2 (2.7%)	4 (8.3%)	0.166
Auto-immune disorder	9 (7.4%)	7 (9.6%)	2 (4.2%)	0.266
**Severity of illness**				
Sepsis-3, sepsis (N, %)	116 (95.9%)	71 (97.3%)	45 (93.8 %)	0.343
Sepsis-3, septic shock (N, %)	12 (9.9%)	6 (8.2%)	6 (12.5%)	0.441
SOFA score (points) (median, IQR)	5 (3–7)	6 (3–7)	5 (3–7)	0.504
**Inflammatory biomarkers**				
WBC, 10E9/L, (median, IQR)	8.3 (6.1–11.8)	8.4 (6.4–12.2)	7.6 (5.4–11.2)	0.317
CRP (mg/L), (mean, sd)	141 (90–205)	138 (90–221)	149 (87–185)	0.592
**Treatment during ICU stay (N, %)**				
High flow nasal cannula	10 (8.3%)	6 (8.2%)	4 (8.3%)	0.982
Invasive mechanical ventilation	111 (91.7%)	67 (91.8%)	44 (91.7%)	0.982
Prone position ventilation	65 (53.7%)	37 (50.7%)	28 (53.8%)	0.409
Corticosteroids (methylprednisolone)	5 (4.1%)	1 (1.4%)	4 (8.3%)	0.060
Vasopressors	99 (81.8%)	61 (83.6%)	38 (79.2%)	0.540
Renal replacement therapy	9 (7.4%)	3 (4.1%)	6 (12.5%)	0.085
**Anti-COVID-19 therapy**				
Chloroquine only	84 (69.4%)	54 (74%)	30 (62.5%)	0.180
Chloroquine + lopinavir/ritonavir	36 (29.8%)	18 (24.7%)	18 (37.5%)	0.131
IL 1 antagonist	2 (1.7%)	0 (0%)	2 (4.2%)	0.079
**Outcome**				
Ventilation in days (median, IQR)	14 (8–23)	14 (7–23)	12 (8–17)	0.945
ICU LOS (days) (median, IQR)	16 (9–31)	18 (9–29)	14 (9–17)	0.621
Hospital LOS (days) (median, IQR)	20 (12–30)	23 (12–33)	17 (10–27)	0.252
28-day mortality (N, %)	34 (28.1%)	19 (26%)	15 (31.3%)	0.532

Legend: All continuous data are presented as median (interquartile range) and categorical data as number (percentage). Abbreviations: COPD: chronic obstructive pulmonary disease, SOFA: Sequential Organ Failure Assessment, WBC: white blood count, CRP: C-reactive protein, IL 1 antagonist: interleukin 1 antagonist, ICU LOS: length of stay in the intensive care unit. Bold values: statistically significant *p* value < 0.05.

**Table 2:** Univariate and multivariate Cox regression 28-day mortality.

Variables			Univariate		Multivariate	
	Patients	Mortality	HR (95% CI)	*p* Value	HR (95% CI)	*p* Value
Age	121	34	1.09 (1.04–1.15)	**<0.001**	1.11 (1.05–1.17)	**<0.001**
Male	121	34	1.45 (0.57–3.52)	0.407	0.84 (0.33–2.13)	0.710
Diabetes	121	34	1.17 (0.54–2.51)	0.697	0.74 (0.32–1.73)	0.493
Hypertension	121	34	1.17 (0.57–2.42)	0.666	1.38 (0.61–3.15)	0.439
SOFA	121	34	1.34 (1.12–1.60)	**0.001**	1.37 (1.12–1.67)	**0.002**
BMI	121	34				
≤30 kg/m^2^			1.0 (Reference)		1.0 (Reference)	
>30 kg/m^2^			1.24 (0.62–2.47)	0.548	1.74 (0.86–3.54)	0.124

Abbreviations: HR: hazard ratio, SOFA: Sequential Organ Failure Assessment, BMI = body mass index. Bold values: statistically significant *p* value < 0.05.

**Table 3:** Univariate and multivariate Cox regression 28-day mortality in males.

Variables			Univariate		Multivariate	
	Patients	Mortality	HR (95% CI)	*p* Value	HR (95% CI)	*p* Value
Age	91	27	1.10 (1.04–1.17)	**<0.001**	1.12 (1.05–1.20)	**0.001**
Diabetes	91	27	1.31 (0.547–2.30)	0.520	0.59 (0.24–1.49)	0.267
Hypertension	91	27	1.44 (0.66–3.14)	0.363	1.70 (0.73–3.99)	0.222
SOFA	91	27	1.33 (1.08–1.63)	**0.007**	1.36 (1.08–1.73)	**0.010**
BMI	91	27				
≤30 kg/m^2^			1.0 (Reference)		1.0 (Reference)	
>30 kg/m^2^			1.10(0.50–2.39)	0.821	1.50 (0.66–3.41)	0.337

Abbreviations: HR: hazard ratio, SOFA: Sequential Organ Failure Assessment, BMI = body mass index. Bold values: statistically significant *p* value < 0.05.

**Table 4:** Univariate and multivariate Cox regression 28-day mortality in females.

Variables			Univariate		Multivariate	
	Patients	Mortality	HR (95% CI)	*p* Value	HR (95% CI)	*p* Value
Age	30	7	1.01 (0.95–1.09)	0.756	1.07 (0.95–1.21)	0.278
Diabetes	30	7	0.55 (0.07–4.58)	0.582	0.97 (0.07–13.33)	0.984
Hypertension	30	7	0.38 (0.05–3.14)	0.367	0.38 (0.03–5.35)	0.476
SOFA	30	7	1.42 (0.99–2.02	0.054	1.33 (0.97–1.82)	0.078
BMI	30	7				
≤30 kg/m^2^			1.0 (Reference)		1.0 (Reference)	
>30 kg/m^2^			2.93 (0.57–15.11)	0.200	4.99 (0.65–38.06)	0.121

Abbreviations: HR: hazard ratio, SOFA: Sequential Organ Failure Assessment, BMI = body mass index.

## Data Availability

Data available upon request.
